# Draft Genome Sequence of Aneurinibacillus migulanus NCTC 7096

**DOI:** 10.1128/genomeA.00234-15

**Published:** 2015-04-02

**Authors:** Faizah N. Alenezi, Hedda J. Weitz, Lassaad Belbahri, Jedidi Nidhal, Lenka Luptakova, Marcel Jaspars, Stephen Woodward

**Affiliations:** a University of Aberdeen, Institute of Biological and Environmental Sciences, Aberdeen, United Kingdom; b Laboratory of Soil Biology, Department of Biology, University of Neuchâtel, Neuchâtel, Switzerland; c NextBiotech, Agareb, Tunisia; d School of Natural and Computing Sciences, University of Aberdeen, Aberdeen, United Kingdom

## Abstract

Aneurinibacillus migulanus has biocontrol activities against fungal, fungus-like, and bacterial plant pathogens with different levels of efficacy depending on the target pathogens. Here, we report the high-quality draft genome sequence of A. migulanus NCTC 7096.

## GENOME ANNOUNCEMENT

Aneurinibacillus migulanus is a Gram-positive, rod-shaped, motile, spore-forming bacterium present in soil environments. An important feature is the production of the secondary metabolite gramicidin S, which inhibits the growth of many microorganisms (1, 2). Because of the ability of A. migulanus to produce antimicrobial compounds, the organism is considered to be a potential biocontrol agent against plant pathogens (1). Many Bacillus species have been tested as biological control agents against plant pathogens as they are less damaging to the environment than chemical alternatives (3–5). Moreover, Bacillus species produce endospores in extreme environmental conditions, an advantage in the long-term storage of Bacillus-based preparations (6). Biological control agents are antagonistic to plant pathogens through various possible mechanisms, including antibiosis, competition for nutrients on infection sites on the plant surface, hyperparasitism, and by induction of host resistance (2, 3). A. migulanus strain NCTC 7096 produces gramicidin S (1).

The genome of A. migulanus NCTC 7096 was sequenced using the BG7 bacterial genome annotation system specifically designed for NGS data (Era7 Bioinformatics, Granada, Spain [7]). Approximately 14.61 million reads were obtained for assembly after low-quality reads were filtered out. The whole genome was *de novo* assembled into 89 contigs (*N*_50_, 157,850 bp) and rearranged into 200 scaffolds.

The draft genome of A. migulanus strain NCTC 7096 comprised 6,270,550 bases with the largest contig of 582,874 bp and 43.16% G+C content. The genome of NCTC 7096 contains sequences encoding 5,130 proteins, of which 1,199 belong to uncharacterized proteins.

### Nucleotide sequence accession number.

The draft genome sequence of A. migulanus NCTC 7096 has been deposited at GenBank under accession no. JYBO00000000. This paper describes the first version of the genome.
